# Health-Related Quality of Life after Pediatric Traumatic Brain Injury: A Qualitative Comparison of Perspectives of Children and Adolescents after TBI and a Comparison Group without a History of TBI

**DOI:** 10.3390/jcm11226783

**Published:** 2022-11-16

**Authors:** Dagmar Timmermann, Ugne Krenz, Silke Schmidt, Michael Lendt, Christel Salewski, Knut Brockmann, Nicole von Steinbüchel

**Affiliations:** 1 Institute of Medical Psychology and Medical Sociology, University Medical Center Göttingen, Waldweg 37A, 37073 Göttingen, Germany; 2 Department Health and Prevention, University of Greifswald, Robert-Blum-Str. 13, 17487 Greifswald, Germany; 3 Neuropediatrics, St. Mauritius Therapeutic Clinic, Strümper Straße 111, 40670 Meerbusch, Germany; 4 Department of Health Psychology, Germany’s State Distance-Learning University Hagen, Universitätsstr. 33, 58097 Hagen, Germany; 5 Division of Pediatric Neurology, Department of Pediatrics and Adolescent Medicine, University Medical Center Göttingen, Robert-Koch-Straße 40, 37075 Göttingen, Germany

**Keywords:** traumatic brain injury, children and adolescents, health-related quality of life, disease-specific assessment, comparison group, qualitative analyses

## Abstract

Background: The assessment of the impact of pediatric traumatic brain injury (TBI) on the health-related quality of life (HRQoL) of the children and adolescents affected can be ameliorated by a disease-specific instrument. Such an instrument does not yet exist. This qualitative study investigates how children and adolescents after TBI subjectively perceive their HRQoL and whether and how this differs from the perspective of individuals without a history of TBI. Methods: Eight problem-centered interviews were conducted with 11 children and adolescents around four years after mild TBI and with eight children and adolescents around three years after moderate to severe TBI. Nine problem-centered interviews were conducted with 25 participants without a history of TBI. The interviews were recorded and transcribed verbatim. The statements were assigned to inductively and deductively derived categories relevant to the HRQoL of children and adolescents after TBI and compared with those of individuals without a history of TBI. Results: The HRQoL of children and adolescents after TBI tended to display both structural and content-related differences, independently of TBI severity, on several HRQoL dimensions, in contrast to the comparison group. For example, participants after TBI reported a broader range of negative emotions (such as worry, sadness, shame, and guilt), permanent physical impairments, felt that they were treated differently from others, and perceived cognitive limitations. Conclusions: The results of this qualitative study identified HRQoL dimensions that are relevant to children and adolescents after TBI and underlined the need for the development of a disease-specific instrument.

## 1. Introduction

Traumatic brain injury (TBI) is the leading traumatic cause of morbidity and mortality in childhood and adolescence in Europe and the US [1]. In Germany, some 81,520 children and adolescents up to the age of 17 suffered a TBI in 2018 [2]. The majority of registered cases in persons under 18 years of age were classified as mild TBI [2]. TBI can be defined as “(…) the consequence of violent impact that has resulted in dysfunction and/or injury to the brain and may be associated with a contusion or injury to the epicranium, the osseous cranium, the vessels, the brain tissue and/or the dura” [3].

Contrary to general opinion, children and adolescents do not always recover well after TBI [4]. It is thought that the degree and persistence of the impact of TBI vary according to severity and age at the time of the accident [5]. The age at which a child experiences a TBI can have a substantial influence on the recovery process. Anderson and Catroppa [4] assume that the less developed specific abilities were at the time of the injury, the more severe deficits may develop.

Outcome measures for pediatric TBI are usually based on physical and neurological functionality [6,7]. With TBI being a very heterogenic and individual injury [8], awareness has increased that TBI can also have a negative impact on emotional, social, behavioral, cognitive, and academic areas [4]. To capture these aspects, the construct of health-related quality of life (HRQoL) provides a widely accepted measure [9,10,11]. Assessment of HRQoL allows the outcome of prevention, treatment and rehabilitation measures for children and adolescents to be evaluated [12], and HRQoL has been established as an important outcome criterion in the pediatric clinical setting [13].

Although there is no consistent definition of HRQoL yet, a broad consensus exists that it is a multidimensional [14] and subjective construct that should, whenever possible, be assessed from the patient’s own perspective [15,16]. Accordingly, whenever children and adolescents are able to provide reliable and valid data, age-adapted self-report measures of HRQoL should be the standard [14,17]. In situations where children and adolescents are unable to self-report, e.g., due to cognitive impairments, it is reasonable to rely on proxy reports [17,18,19]. However, for parent proxy reports, potential discrepancies with children’s HRQoL reports must be considered. Several studies have shown that parents of ill children rated the HRQoL of their children lower than the children themselves, whereas this discrepancy was reversed in non-clinical samples [20,21].

Essentially, two classes of instruments may be used to assess HRQoL: cross-disease (generic) and disease-specific instruments. Generic instruments provide an overview of the perceived health, health behaviors, and subjective well-being and allow subjective health status to be compared across different diseases [16,22]. These are suitable for studies in which the HRQoL of children and adolescents with different baseline conditions (e.g., different diseases) or interventions are compared [23]. However, especially in the clinical context, disease-specific instruments are particularly suitable, as they facilitate the assessment of symptoms or other HRQoL factors relevant to the respective patient population [14,23,24,25]. In contrast to generic instruments, the latter is more sensitive to the specificity of the disease. They may therefore be more effective when assessing the actual subjective health status or the health status of a child or adolescent after having received a type of treatment and adjusting this as needed in individual cases [14,23,24,25]. 

As there is currently no disease-specific instrument for assessing TBI-specific HRQoL in children and adolescents in an age-appropriate manner from 5 to 17 years of age as well for their parents, the QOLIBIRI-Kiddy/Kid/Ado project aims to develop the first instrument for the assessment of disease-specific HRQoL in children and adolescents who have suffered a TBI. 

When developing instruments for the assessment of HRQoL, Coghill et al. [26] recommend a combination of literature research and expert interviews, as well as the use of a survey with patients as a central component. Langlois [27] points out that qualitative research offers a good opportunity to gain a better understanding of the experiences from the point of view of children and adolescents after TBI. 

In the present study, the method of semi-structured interviews was used to determine relevant aspects of HRQoL from the perspective of children and adolescents. The primary aim of this study was to learn from the affected individuals about their subjective perspectives on their HRQoL after TBI as a prerequisite for the development of a TBI-specific HRQoL instrument and to explore whether and to what degree the subjective perspectives on HRQoL of children and adolescents after mild, moderate to severe TBI differ from those of individuals not affected by TBI. In addition, differences between children and adolescents after mild TBI (mTBI) and moderate (mod) to severe (s) TBI (mod/sTBI) were considered on the assumption that relevant contents of HRQoL after TBI may vary according to severity.

## 2. Materials and Methods

The present study was approved by the ethical committee of the University Medical Center Goettingen (UMG), Germany (file nr. 21/3/14). After the participants had been informed in detail about the aim and design of the study, written informed consent was obtained from all of them.

### 2.1. Design

To determine the views of children and adolescents about their HRQoL, we applied a qualitative approach using problem-centered interviews, as proposed by Witzel and Reiter [28]. This type of interview is considered a suitable method since it encourages the researcher to mediate between existing empirical knowledge and future knowledge to be explored. As a result, researchers may be more attentive to what participants report, and data collection is influenced less by the researcher’s own perceptions and experiences. A semi-structured interview guideline was used, covering previous theoretical considerations on general and TBI-specific HRQoL. As the interviews were designed as an open, moderated, problem-centered discussion in small groups, it was also possible to encourage participants to explicate and/or develop their own perspectives on a topic in a narrative way. These perspectives were followed up by the interviewer on an ad hoc basis to initiate a dialogue that complemented and expanded our previous theoretical assumptions. Information on TBI severity and fulfillment of the inclusion and exclusion criteria were extracted from medical records by the staff in the recruiting centers. Age and accident data were obtained through self-reports by participants and/or parents or legal guardians.

### 2.2. Recruitment of Participants

With the approval of the ethics committee of the University Medical Center Goettingen (UMG), Germany, 20 children and adolescents with a history of TBI were recruited retrospectively by the medical staff of the cooperating departments and clinics (Clementine Kinderhospital; Department of Health and Prevention, University of Greifswald; Department of Pediatrics and Adolescent Medicine, Division of Pediatric Neurology, UMG; Neuropediatrics, St. Mauritius Therapeutic Clinic). The participants of the comparison group (*n* = 27) were identified via postings in local nursery schools and the social networks of the employees of the Institute of Medical Psychology and Medical Sociology of the UMG (IMPIMS/UMG). 

Individuals between five and 17 years of age who had a good command of the German language were included, and informed consent forms were obtained both from them and from their parents. Further requirements for the TBI sample included an ICD-10 diagnosis of head injury (S06.0–S06.9), an out-patient status, and the elapsed time since the TBI should be between three months and ten years. Exclusion criteria for the comparison group comprised having a history of TBI. Subjects in both samples were excluded if they fulfilled one or more of the following criteria: spinal injuries, severe mental illnesses (e.g., psychosis, etc.) and autism, serious substance addiction, and/or lethal diseases. Moreover, we excluded individuals from the study if they were incapable of understanding and cooperating (e.g., because of severe cognitive impairments, etc.). 

The current manuscript focuses on the differences between the HRQoL of children and adolescents with and without a history of TBI.

### 2.3. Procedure

Participants with and without a history of TBI were predominately interviewed in separate small groups divided into three age groups (5 to 7 years, 8 to 12 years, and 13 to 17 years). The interviews took around 60 to 90 min and were conducted by a principal and an assistant moderator.

Each interview consisted of two parts. In the first part, a semi-structured interview guideline was used, covering previous theoretical definitions of generic and TBI-specific HRQoL, among other aspects. TBI-specific questions were adapted for the participants without a history of TBI in the comparison group so that they were asked to answer the questions related to an accident or prolonged illness they had experienced. The technique of open interviews makes it possible to encourage participants to explicate and develop their own perspectives on the topic in a narrative way. In the second part, participants were presented with an age-adapted version of the QOLIBRI for adults [29,30] to obtain information regarding age-appropriate item content, wording, comprehensibility, the relevance of the items, and the suitability of various response formats.

All interviews were conducted face-to-face in quiet rooms. Seven interviewers (six female and one male) collected data from the participating children and adolescents. The moderators of the interviews held at least an undergraduate qualification, e.g., a bachelor’s degree in Psychology, and were introduced to the technique of semi-structured interviews and briefed on the interview guideline by associates of the IMPMS/UMG. There had been no previous contact with the children and adolescents interviewed. 

Eight problem-centered interviews were conducted with children and adolescents after mild, moderate, and severe TBI (participants per interview: *Mean* = 3, *Range* = 1–5). Nine problem-centered interviews were conducted with children and adolescents without a history of TBI (*Mean* = 3, *Range* = 2–6). One interview with a participant from the TBI sample was conducted as a single discussion. 

### 2.4. Data Analysis

All interviews were recorded and transcribed verbatim. The program MAXQDA (v.12) was used for the data analyses. First, data were subjected to a qualitative content analysis according to Mayring [31], a method suitable for working on transcripts of interviews [32]. Even though a general model is provided by Mayring, the content analyses should be adapted to the particular data [31]. For the qualitative analyses, several techniques can be applied [32]. Within our study, a combination of inductive (bottom–up) and deductive (top–down) content structuring was used. For deductive category development, categories are defined prior to the analyses [33]. This procedure is suitable for semi-standardized interviews using, e.g., a theory-based interview guideline [34]. The inductive category development, on the other hand, is especially suitable for free exploration [34]. Here, categories are derived from the raw material [33], in our case, more than 500 pages of literally transcribed interview text, by combining statements that are related to each other or aspects of the same content into one category. The additional inductive procedure allowed aspects to be detected that had not been captured by the theory-based deductive approach. Three coders independently carried out the content analyses. In an iterative-analysis process, a final coding guide was developed with definitions, anchor examples, and coding rules for each theory-based and additional category. The purpose of this process was to generate an exhaustive and situated system of categories [35]. 

Based on the general process model, according to Mayring [31,32], in the first step, approximately 40% of the original data on TBI participants was analyzed. Statements were either deductively assigned to the theory-based categories covered by the interview guideline or initially assigned to a residual category if the statements were not captured by the pre-assigned categories. Accordingly, the residual category comprised all statements with HRQoL aspects, from which new categories were later inductively derived in the following step. In the second step, the categories were specified: definitions, anchor examples, and coding rules for the deductive categories were revised. Inductive categories were generated by summing up the content of the statements initially assigned to the residual category, which were then specified by definitions, anchor examples, and coding rules. In a third step, transcripts of the interviews were inductively and deductively analyzed separately for children and adolescents after TBI and for those from the comparison group. After all the interviews had been analyzed, the fourth step was (a) to summarize the categories resulting from the separate group analyses and (b), based on theory, to assign them to the six dimensions of the HRQoL model according to von Steinbüchel et al. [29,30] and a supplementary Covariates category. The fifth step compromised re-coding the entire data material. In the sixth step, an independent consensus check was carried out by three independent raters, and intercoder reliability was determined for all dimensions by pairwise comparison. The initial agreement, which provided the basis for the final consistency check, was considered satisfactory, with an average agreement of 84%. In the final step, all disagreements were analyzed and discussed until consensus was achieved, resulting in a finalized system of categories and data for further analysis.

For the current study, we additionally analyzed statements assigned to the six dimensions of HRQoL using a case-based method [34] to examine differences and similarities between TBI severities and the comparison group in more detail. Instead of analyzing the statements of participants individually, all participants of the respective groups (mTBI, mod/sTBI, and CG) were considered and analyzed as one case on the group level. The case-specific analyses of the statements within a category were based on the concept of open coding in grounded theory [34] and the summarizing of qualitative content analyses, according to Mayring [31]. This approach additionally focuses on concepts associated with the coded statements within a category. As the primary focus of this study was to explore core aspects of self-reported HRQoL in children and adolescents, statements coded to the supplementary category Covariates were not included in the further analyses.

Because of the small sample size, further differentiation into age groups was not considered useful for exploring the research question of the present study. The results of the qualitative analyses focusing on age groups in the entire sample of children and adolescents after TBI have been reported previously [36].

Due to the qualitative design and exploratory nature of the present study, the results of the analyses are primarily presented descriptively. However, to provide an initial overview of the assigned statements across the six theory-based dimensions and to permit a comparison between the groups, the relative frequencies per group are presented.

## 3. Results

Three participants were excluded due to violations of inclusion and/or exclusion criteria, and the final sample consisted of 19 participants after TBI (11 mTBI, five moderate and three severe TBI) and 25 participants in the comparison group without TBI. To determine whether the data from the participant interviewed alone could be included in the analyses, the number of coded statements was compared with the mean number in the corresponding age group after TBI. Since the number of statements was in the middle of the group range (19 coded statements, *M* = 13.6, 95% CI [2.73, 24.47]) covering a wide range of contents, the data for this individual interview were included in the analyses.

The groups of children and adolescents after moderate and severe TBI were aggregated into one group because of the small number of participants. We considered pooling the two groups acceptable as, e.g., McCarthy et al. [37] reported comparable declines in domains of HRQoL, particularly for children and adolescents after moderate and severe TBI. Table 1 provides an overview of the sociodemographic data of interviewed children and adolescents after mTBI, mod/sTBI, and for those from the comparison group.

**Figure 1:**
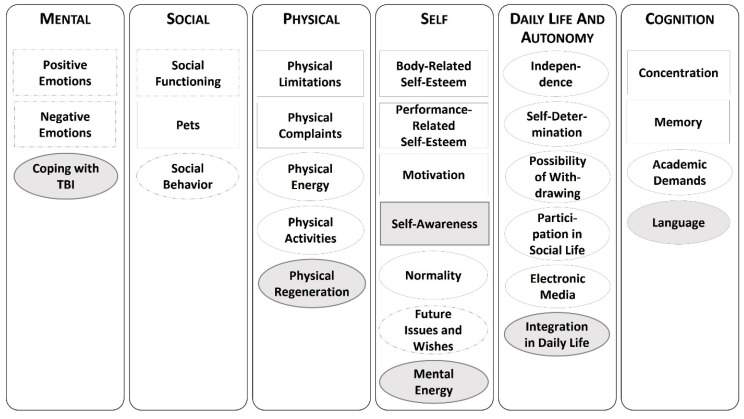
Category system. Note: Deductive categories are shown in rectangles, and inductive categories are in ovals. Gray-shaded categories contain only statements of children and adolescents after traumatic brain injury (TBI); categories with dashed outlines are further specified by subcategories.

In line with the exploratory qualitative design, the final coding scheme was very detailed. Figure 1, therefore, provides an overview of the main categories assigned to the theoretically based HRQoL model.

As can be seen in Figure 1, some of the categories were further specified by subcategories and will therefore be described in more detail here. In the category of *Positive Emotions*, a further distinction was made between statements related to *Being Happy/Having Fun*, *Enjoying Life*, or *Feeling Safe and Secure*. The category of *Negative Emotions* included the following subcategories: *Worries*, *Anxiety*, *Sadness*, *Shame*, *Anger*, *Guilt*, *Stress*, and *Boredom*. Within the category of *Future Issues and Wishes*, a distinction was made between *Plans* (e.g., about the academic career) and *Wishes* (e.g., referring to oneself, to relevant social others, or material things). The category *Social Functioning* captured statements related to *Family*, *Friendships,* and *Kindergarten*/ *School*. The category Social Behavior comprised the following subcategories: *Social Behavior of the Child*, *Problem Behavior of the Child,* and *Pets.*

**Figure 2:**
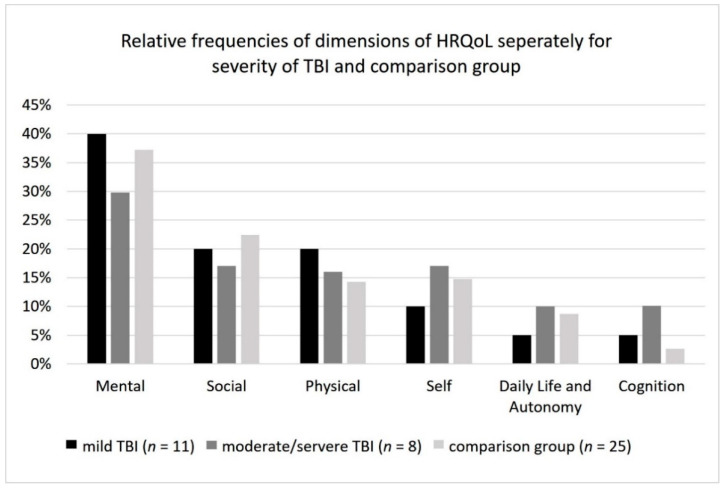
Relative frequencies of main dimensions of health-related quality of life (HRQoL) for mild traumatic brain injury (TBI), moderate to severe TBI, and comparison group.

Overall, 178 statements of children and adolescents after mTBI, 190 of those after mod/sTBI, and 379 from the comparison group were coded on six model-based dimensions from the QOLIBRI questionnaire by von Steinbüchel et al. [29,30], which together comprised 28 categories. Figure 2 shows the relative frequencies of statements coded on the dimensions of HRQoL reported separately for the two TBI groups and the comparison group.

The results of the qualitative analyses will now be presented individually for each of the six dimensions. Core findings are additionally supported by anonymized sample quotes from the original interviews. 

### 3.1. Mental Dimension

This dimension includes all statements related to positive or negative emotions as well as coping psychologically with the accident and the TBI.

Across all groups, positive emotions were mentioned, especially in connection with physical activities, sports, and joint activities with one’s own family (e.g., “Spending time doing something or spending time doing what you love or also doing something with people you like. In my opinion that is what makes me happy, too.”) Participants of the comparison group and after mTBI also reported positive emotions related to school or kindergarten as well as spending time with their friends. Children and adolescents after mod/sTBI, on the other hand, stated that they were happy if they had enough freedom and leisure time to do what they wanted. Statements by children and adolescents of all severities that reflected aspects of HRQoL or well-being were mainly related to enjoying life (without restrictions) and taking full advantage of life. (e.g., “On the one hand, love, but also thrills. Doing something new and gathering experience. I think that’s what makes sense in life, to gain experience”). In contrast to this, statements by the comparison group primarily referred to the fact that they saw their current life as being perfect the way it was. Statements by all participants after TBI reflected a wider range of negative emotions than those by participants from the comparison group. These statements referred to feelings such as worries, sadness, shame, and guilt. Especially for children and adolescents after mod/sTBI, negative emotions were often mentioned in connection with the causes and consequences of TBI/ the accident. For example, they reported mourning relatives who had died in the accident or sadness caused by thoughts about the accident. (e.g., “Sometimes I still come across these situations where I, I don’t know, where it [i.e., the accident] just makes me a little sad again, […]”) Comparing the groups also revealed that more statements associated with negative emotions were made by children and adolescents after mod/sTBI than by those after mTBI and by the comparison group. In all TBI groups, participants reported that they had not completely overcome the accident mentally. Especially children and adolescents after mTBI reported more cautious or avoid behavior since the accident (e.g., “[…], that I always try not to let a ball hit my head because I still remember the impact. And it was rather painful.”).

### 3.2. Social Dimension

All aspects of social functioning in the context of family, friends, the school environment, and self-reported social behavior of the children and adolescents themselves were included here. 

In all groups, participants reported that they enjoy spending time with their families, that their families are important to them, and that family members act as relevant persons of trust. In addition, children and adolescents after TBI mentioned that they miss their families when they are separated from them. For all groups, friendship includes statements that friends play a central role in their lives. Participants after TBI expressed the importance of supporting each other or getting along well with one’s friends. Participants after mod/sTBI also mentioned that people in their environment had turned out to be false friends or had no best friend at all (e.g., “Yes. I do many things together with him/her [pseudonym], he/she [pseudonym] is a good friend of mine, but not a best friend. I don’t have any best friends.”). Only participants from the comparison group and the mTBI group explicitly stated that they enjoyed going to school. Regarding the social dimension, participants in the comparison group and the mod/sTBI group reported socially non-conformist behavior, such as not letting other children play with them or being involved in fights. In addition, participants after mod/sTBI also reported phases of anger in which they tended to become physically aggressive toward others (e.g., “Sometimes I also freak out, then I just hit him/ her [pseudonym]. But not too hard since he/she [pseudonym] is my buddy.”).

### 3.3. Physical Dimension

This dimension comprises all statements reflecting physical activity, physical limitations, physical complaints, pain, physical energy as well as physical regeneration.

Everyday physical activities during school breaks or on playgrounds tend to play a similarly central role for participants in all groups. Regardless of the severity of TBI, participants reported permanent physical limitations, such as coordination disorders that have a negative impact on their everyday activities (e.g., “I like to run around and playing tag. Better not playing tag, I will fall over. Just running around to get faster.”). However, the physical limitations reported by the comparison group were mainly related to acute injuries or diseases. (e.g., “In my case, my $$sensitive text passage## was almost broken […] and I couldn’t play $$sensitive text passage## anymore.”) Regardless of TBI severity, most physical symptoms and pain reported can generally be described as chronic. A common type of pain reported was that of regularly recurring headaches (e.g., “With me, it was like that, after the accident and still now, I sometimes have headaches and I don’t feel so well.”). Additionally, in the mod/sTBI group, permanent back pain and discomfort in scar areas were mentioned, and in the mild TBI group, constantly recurring nausea. Participants in the comparison group, on the other hand, talked about acute pain and physical symptoms (e.g., “Whenever I get injections, […], my arm hurts, and even now it is still hurting a little bit, I still can’t move it.”). Additionally, children and adolescents after mTBI felt restricted in their everyday lives because of increased fatigue since their accident (e.g., “I didn’t feel good. I just wanted to go to bed, and I was tired all the time.”). The feeling of being nearly completely or partially physically regenerated was reported by participants regardless of TBI severity (e.g., “Actually, I was still the same person, except that I had headaches all the time and things like this. I was like, it just followed me. […], but now everything is under control.”).

### 3.4. Self Dimension

Statements related to self-esteem, motivation, self-confidence, mental energy, future aspects, plans, and wishes are reported here.

In all groups, most of the statements referred to the participants’ own future perspectives and their plans for life (e.g., school career, training, and academic training). The differences and similarities identified between the groups can be described as cross-group and interest- or age-dependent. Asked about wishes for the future, most of the statements made by the comparison group concerned wishes directly related to themselves (e.g., own health, satisfaction, and continuity). Especially for participants after TBI, it was important to regain more self-confidence (e.g., “Yes, that I just no longer, that I regain more self-confidence and that, I am not as calm at home, but at school, I have just become calm and reserved.”) Additionally, participants of this group perceived a different, more negative physical self-esteem after TBI (e.g., “I don’t like it. I myself can touch it [i.e., the scar], but if others just touch it without me knowing about it, I really don’t like this at all.”) Regarding performance-related self-esteem, the statements by children and adolescents from all groups were primarily related to satisfaction or dissatisfaction with their academic performance at school. Regarding motivational aspects, qualitative differences in content were found between the TBI and the comparison group. While participants after TBI expressed that they were tackling challenges and pursuing their own goals, the comparison group reported having problems motivating themselves sufficiently or a tendency to postpone things (e.g., “Well, with me, it’s like this, I get stressed because I always postpone everything and then I do something other than studying, for example, and then it’s always like this in the evening.”) Concerning the aspect of normality, participants after TBI perceived that people from their social environment often focused on their accident and the resulting changes, e.g., that they were preferred by teachers because of the TBI. In contrast, statements from the comparison group primarily referred to social comparisons of their own abilities or skills with those of their peers. Furthermore, participants after mod/sTBI reported that they often felt mentally exhausted after school (e.g., “It’s stupid. I don’t have any time. Just homework, eat, sleep. […] I’m so knocked out.”).

### 3.5. Daily Life and Autonomy Dimension

These statements reflect aspects of participating in social life, electronic media, orientation, the possibility of backing out, independence, self-determination as well as the process of reintegration into daily living.

Participating in leisure activities such as sports, dancing, and/or singing was found to be one major aspect across all groups. No differences were identified between the groups regarding their use of media for entertainment or keeping themselves occupied, e.g., watching films, television, YouTube, or playing digital games. The need for rest and time for oneself was also reported by all groups. Concerning the issue of self-determination, especially the comparison and mTBI group reported that they considered themselves to have sufficient personal freedom. Being able to do and undertake things independently without help from others was primarily addressed by the comparison group. Especially children and adolescents, after mod/sTBI talked about the period of reintegration into their social environment following the TBI/accident. Statements mainly referred to experiences in the context of school (e.g., “There was, first of all, a phase of getting used to it. I still remember, at first, I was there [i.e., at school] on one day for two hours […]. After that, I was at school the whole week for six hours, and at some point, I had school in the afternoon, and then it worked out again.”).

### 3.6. Cognition Dimension

Here, statements concerning aspects of academic performance, concentration and attention, memory functioning as well as producing and understanding (spoken) language are classified. 

While participants after mod/sTBI were more likely to report that they were able to meet the academic demands of school, the mTBI group experienced difficulties keeping up with the academic performance of their classmates. Feeling a pressure to perform and a sense of inner restlessness before exams were discussed only by participants after mTBI and the comparison group. On the other hand, aspects concerning concentration and attention were primarily mentioned in the group after mod/sTBI. While some participants explicitly emphasized having no problems maintaining concentration over a prolonged period, others reported increasing difficulties concentrating since the injury. Whereas the group after mTBI did not mention any aspects related to concentration, the comparison group reported being easily distracted. Regarding memory performance, both TBI groups perceived limitations in the form of memory lapses, which they noticed only after the TBI (e.g., “For example, I always used to be able to remember what happened two days ago, but now I just don’t remember anything anymore.”). Concerning speech and understanding, only participants after mod/sTBI reported difficulty in finding words and reading.

## 4. Discussion

The main objective of the current study was to explore whether and to what extent differences exist between self-reported aspects of HRQoL of children and adolescents after TBI compared to those without a history of TBI. The qualitative analyses revealed that the TBI group reported a broader range of aspects concerning HRQoL than the comparison group. The additional aspects (e.g., negative emotions such as sadness and shame, reduced self-esteem after TBI, cognitive fatigue) reported by the TBI groups, which can be interpreted as especially relevant to the phenomenon of TBI, provide important information for constructing a disease-specific questionnaire to assess HRQoL.

The qualitative analyses of the mental dimension of HRQoL showed that, particularly for negative emotions, the TBI group reported a broader range than participants from the comparison group. Especially in the group with mod/sTBI, these were mentioned in the context of the causes and consequences of TBI. These findings are in line with previous studies which found TBI to be a risk factor for emotional problems or even disorders [5,38,39,40,41,42]. Moreover, Glang et al. [43] point out that negative feelings over lost skills are often not adequately addressed and can result in long-term detrimental effects on the perception of the self-concept. It should be noted that causes of changes in the emotional state can be either cerebro-organic or reactions to changes and consequences after TBI, which should be treated differently [44]. 

According to Anderson and Catroppa [4], reintegration into daily life is a central and final component of rehabilitation. This includes the resumption of activities in social networks. Since social and behavioral problems are quite common after pediatric TBI, reintegration into previous social life and activities can be another challenge for those affected [45,46,47]. A systematic review of social functioning in children and adolescents after TBI indicated that especially children after mod/sTBI have an increased risk of developing social problems, including fewer close friendships [48]. The results of more recent studies, however, provide evidence that social-emotional problems occur independently of TBI severity [49,50]. In our study, statements about having no friends or no reliable friends were only found in the mod/sTBI group. Additionally, periods of anger were reported during which participants were no longer able to control their behavior. Changes in personality were also observed in about 10 to 20% of children after TBI, most often accompanied by unstable, aggressive, and—in terms of kneejerk reactions—disinhibited behavioral changes [51].

Regarding the physical dimension, another key finding of the comparative qualitative analyses was that participants in both groups after TBI tended to report chronic complaints and limitations, whereas the comparison group tended to report more acute health conditions. In both TBI groups, participants frequently reported suffering recurrent headaches, which is considered a common somatic problem after pediatric TBI [52]. Additionally, in the group of mod/sTBI permanent back pain and discomfort in scar areas were mentioned, and in the mTBI group, constant recurring nausea. Tham et al. [53] have demonstrated that persistent pain, also in anatomic regions other than the head, is a common issue after TBI with a negative effect on HRQoL. The presence of pain after pediatric TBI was also found to be an important risk factor for depressive symptoms [54].

Statements describing the self-perception of participants after TBI referred to reduced body-related self-esteem, mainly associated with accident-related physical changes. Furthermore, the TBI groups reported reduced self-esteem after the accident, which was mainly associated with the perception of an altered personality. The preceding results are consistent with the findings of other studies. For instance, Hawley [55] showed that pediatric patients after TBI reported lower self-esteem compared to a control group of participants without a history of TBI or neurological impairments. According to Glang et al. [43], perceptions of differences in self-concept due to the accident lead to a developmental process in the search for a new identity up into adulthood. 

In contrast to several previous findings reported in the current literature [38,43,56], participants after TBI in the present study did not report a loss of motivation but, on the contrary, indicated that they were tackling challenges and pursuing their own goals. This could be explained by the disability paradox, which states that individuals with a disability tend to rate their HRQoL on the same level as or even higher than people without a disability [57,58]. Based on the findings of McFarland and Alvaro [59], another possible explanation for this finding may be that the experience of suffering a TBI may reinforce coping with perceived physical and mental limitations.

Additionally, participants in the mod/sTBI group stated that they felt mentally exhausted, which may correspond to the syndrome of cognitive fatigue [60]. 

Another aspect of particular importance for children and adolescents after mod/sTBI was reintegration into their social environment, especially returning to school. Although not all children and adolescents need special academic support after TBI, a guided reintegration into school can be useful for most of them, taking into account the different phases after TBI [61]. It is extremely important to provide relevant information concerning long-term cognitive, behavioral, and/or social deficits to the relevant persons in school [47] since a lack of information may hinder a successful school outcome [43].

Memory lapses, difficulty concentrating, and difficulty in finding words was mainly discussed by the group after mod/sTBI. In apparent contradiction to this, participants after mod/sTBI reported being able to cope with academic demands, whereas children and adolescents after mTBI perceived having problems catching up with the academic performance of their classmates. This finding may also be associated with the aforementioned disability paradox. Even though children and adolescents, after mod/sTBI, reported changes in cognitive aspects, they might underestimate the impact of those changes on their academic performance. Several studies are available to date in which cognitive functioning and school performance of children and adolescents after TBI are investigated. While the long-term recovery of cognitive functioning after mTBI is controversial in the literature [62], studies of neurocognitive outcomes after severe TBI indicate significant impairments regarding attention, processing speed, and delayed memory, among others [6]. A meta-analysis that focused on investigating impairments in academic domains of reading, math, and spelling after pediatric TBI revealed that children after mod/sTBI had persisting deficits, whereas neither post-acute nor chronic deficits were found in children after mTBI [63]. According to the authors, the fact that differences in academic skills become apparent later than in neurocognitive functioning can be ascribed to the fact that neurocognitive functioning provides the basis for academic learning. It can therefore be assumed that deficits in neurocognitive functioning have a cumulative effect on the development of academic skills over time [63]. Another possible reason why children and adolescents do not perceive problems meeting school requirements after TBI could be that academic demands have been adjusted to meet the child’s needs, such as being transferred to another school or repeating a grade.

In summary, the detailed case analyses of the interviews showed that the dimensions of HRQoL in children and adolescents after mild, and to an even greater extent, after mod/sTBI are characterized by a different and broader range of impairments and restrictions than in the comparison group. In their scoping review on neurotrauma centers, Dasic et al. [64] demonstrated that to date, there are still manifold unmet needs in the field of TBI, as the assessment of patient-reported outcomes (including HRQoL), a lack of rehabilitation and follow-up services, especially in low- and middle-income countries. Therefore, we presume that an assessment of HRQoL that considers TBI-specific aspects will prove particularly valuable in rehabilitative care and medical/ psychological aftercare.

### 4.1. Limitations

The results of the analyses based on an explorative qualitative approach can provide initial indications of possible differences in HRQoL between children and adolescents after TBI and those from a comparison group. As reported in the literature, moderated small group interviews in pediatric health care research provide a useful methodology to depict children’s views on health-related topics [65,66]. Yet, a potential limitation of the method could be that the responses of participants may have been influenced by group consensus, social pressure, or favoring more talkative or dominant participants [67,68]. By creating a comfortable, supportive, and open conversational atmosphere, it yet seemed possible to establish a valuable, equally distributed, and stimulating interaction between the participants. 

Due to the chosen qualitative design and the correspondingly small sample, trends and contents cannot be generalized. Regarding the comparison group, it should be noted that these participants came predominantly from the social network of UMG employees and, therefore, may overrepresent children and adolescents from academic households. In addition, in terms of the analyses of the differences between the groups, data for the respective groups were aggregated by age and sex. Therefore, no conclusions can be drawn based on these variables. 

As the investigation of sex differences was not in the scope of the current study, the different and unequal distribution of sex in the TBI groups, in contrast to those of the CG, may present a further limitation. We, however, believe that a different sex distribution would not have had a relevant impact on the breadth of aspects reported in the groups since they are in line with findings from the cited studies. Yet, it may nevertheless have potentially influenced the reported relative frequencies of statements. A recent study on sex differences [50], e.g. suggested lifelong differing psychological outcomes concerning internalizing problems (e.g., depression and anxiety) and externalizing problems (e.g., substance abuse) in males and females after childhood TBI and a control group. Females reported significantly more internalizing problems, and males significantly more externalizing problems. Although our sample is not representative regarding the distribution of sex, we are convinced on having collected a sufficiently great variety of statements from both sexes. Potentially, the overrepresentation of female participants in the TBI groups in our study may also be considered an advantage of the current study, as females are often underrepresented in TBI research due to higher incidence rates in males [50].

Another limitation concerning the generalizability of the reported results may be associated with the time since injury. Participants with a shorter time since injury, e.g., six to twelve months or less, may have reported more severe impairments of their HRQoL. From TBI literature, one knows that most changes (i.e., ameliorations) in HRQoL occur after six months [12]. However, in the current study, despite the relatively high mean time since injury, manifold limitations with respect to all dimensions of HRQoL were reported by children and adolescents after TBI. In our opinion, the findings underline the relevance of assessing TBI-specific aspects of HRQoL not only in the acute phase but several years post-TBI.

### 4.2. Implications and Further Research

Despite the limitations mentioned above, the results of the qualitative analyses of the problem-centered interviews confirm that constructing a TBI-specific instrument is justified. The detailed qualitative comparison of the statements showed that the dimensions of HRQoL of children and adolescents after TBI were characterized by a broader range of aspects compared with the comparison group, especially for individuals after mod/sTBI. The inclusion of affected persons in the process of constructing a disease-specific HRQoL instrument is therefore relevant: it allows researchers to detach themselves from their own view of the ‘case’ and theoretical knowledge and to recognize children and adolescents as experts on their own self as a central source of relevant and unknown information.

The results of the qualitative analyses showed that for children and adolescents after TBI, in addition to general aspects of pediatric HRQoL mentioned in all groups, aspects such as physical regeneration, coping with TBI, regaining self-awareness, fatigue, reintegration into their social environment and memory performance after TBI, were relevant. We, therefore, assume that a disease-specific instrument that measures these relevant aspects of HRQoL after pediatric TBI will lead to a more comprehensive and sensitive assessment than a generic one, especially regarding the evaluation of medical and/or psychological care and rehabilitation after TBI. 

The dimensionality of HRQoL, according to von Steinbüchel et al. [29,30], proved to be a suitable framework model for structuring the categories determined through the content analyses. However, only by testing and determining the psychometric quality criteria of the items is it possible to determine whether and to what extent the assignment of the categories to those scales is meaningful.

## Figures and Tables

**Table 1:** Demographic characteristics of participants.

	mTBI Group *n* = 11	mod/sTBI Group *n* = 8	Comparison Group *n* = 25
Mean age at assessment	*M* = 10.75 (*SD* = 3.62)	*M* = 12.38 (*SD* = 2.88)	*M* = 11.15 (*SD* = 4.25)
Sex	8 female 3 male	5 female 3 male	9 female 16 male
Mean time since injury (in years)	*M* = 3.88 (*SD* = 2.51) *	*M* = 3.25 (*SD* = 2.31)	------------------

Note: TBI = traumatic brain injury, mTBI = mild TBI, mod/sTBI = moderate to severe TBI, *n* = number of participants in the subsample, *M* = mean, *SD* = standard deviation. * *n* = 10.

## Data Availability

Data presented in this study are available only in an anonymized and aggregated form with anonymized examples for the categories upon request from the corresponding author. Relevant anonymized data are provided within the manuscript. The fully transcribed interviews are unavailable to publications and third parties due to ethical restrictions determined by the Ethical Committee of the University Medical Center Göttingen (UMG), Germany.
